# Family cohesion and adolescent self-esteem: mediating effects of interpersonal self-support and moderating role of school attachment

**DOI:** 10.3389/fpsyt.2026.1828156

**Published:** 2026-10-27

**Authors:** Zhendong Yao, Yuhang Luo

**Affiliations:** 1 College of Educational Sciences, Hunan University of Arts and Sciences, Changde, Hunan, China; 2 College of Educational Sciences, Hunan Normal University, Changde, Hunan, China; 3 No. 2 Middle School of Linli County, Changde, Hunan, China

**Keywords:** adolescent, family cohesion, interpersonal self-support, school attachment, self-esteem

## Abstract

**Introduction:**

Adolescent mental health is a pressing global concern, and in collectivist societies such as China, family and school contexts are particularly influential. This study examined whether interpersonal self-support mediates the association between family cohesion and self-esteem among Chinese adolescents and whether school attachment moderates the first stage of that mediation pathway.

**Methods:**

A cross-sectional survey was conducted with 728 adolescents (mean age = 13.81 years, *SD* = 0.91, range 12–16; 44.0% male) from five public junior high schools in Hunan Province, China. Confirmatory factor analyses were conducted in Mplus 8.3, and the moderated mediation model was tested with Hayes' PROCESS macro (Model 7) in SPSS 27 using observed variables and 5,000 bootstrap samples.

**Results:**

Family cohesion was positively associated with self-esteem and with interpersonal self-support, and interpersonal self-support partially mediated the family cohesion–self-esteem association. School attachment moderated the first stage of the mediation pathway, *b* = 0.076, 95% CI [0.024, 0.128], Δ*R*^2^ = .008: the positive association between family cohesion and interpersonal self-support was significant at all levels of school attachment and was stronger among adolescents reporting higher school attachment, and the conditional indirect effect increased from 0.107 at low to 0.153 at high school attachment.

**Discussion:**

The indirect pathway was secondary to the direct association between family cohesion and self-esteem, and the moderation effect was modest. Because the design was cross-sectional and self-reported, these associations should not be interpreted causally. The findings suggest that schools may support the benefits of family cohesion for adolescent self-esteem by strengthening teacher-student relationships and cultivating a sense of belonging.

## 1 Introduction

In contemporary society, adolescent mental health has become a concern that cannot be ignored. According to the Report on the Development of Chinese National Mental Health (2023–2024; 1), the risk detection rate of depression among adolescents is as high as 26.1%, markedly higher than that of other age groups. This phenomenon may be associated with adolescent self-esteem levels. As a core constituent of self-concept, self-esteem encompasses individuals’ cognitive-affective evaluations of personal competence and intrinsic worth (2). Given its empirically established associations with psychological well-being and psychosocial adaptation (3), diminished self-esteem appears as a clinically observable correlate in adolescents experiencing depressive symptomatology, typically deteriorating in tandem with depressive episode severity. Investigating the mechanisms underlying self-esteem development in adolescents therefore has significant practical implications.

Adolescent self-esteem is shaped by the growing environment, and studies indicate that family factors are closely related to self-esteem development (4, 5). The family operates as society’s core structural foundation and as the principal ecological framework for adolescents’ biopsychosocial maturation. This multifaceted construct integrates evaluative dimensions of family functioning, spanning structural composition, adaptive capacities, environmental characteristics, and systemic functionality. As a key dimension of family functioning, family cohesion refers to the degree of emotional connection between family members (6). Poor family cohesion has been linked to various psychological problems and more problem behaviors in teenagers (7), whereas good family cohesion is associated with enhanced self-regulation and academic achievement (8). Given these associations, the present study focuses on the relationship between family cohesion and self-esteem.

While prior investigations have predominantly focused on direct associations between family cohesion and self-esteem (9, 10), critical gaps remain in understanding the underlying mechanisms. Existing paradigms often overlook intrapersonal and interpersonal mediators, particularly the construct of interpersonal self-support, a critical dimension of autonomous personality development. Defined as the integrative cognitive-behavioral capacity cultivated through self-regulated resolution of interpersonal challenges, this trait encompasses multifaceted personality dispositions with a distinct relational orientation. Empirical evidence indicates that adolescents exhibiting higher interpersonal self-support demonstrate better applied problem-solving competencies, suggesting its potential role in translating familial cohesion into psychosocial resilience (11). Furthermore, individuals with higher interpersonal self-support tend to report higher self-esteem, although this relationship may vary by age and sociocultural context (12). At the same time, school is a primary activity setting for teenagers, and the school environment may also influence adolescents’ development. Consequently, this investigation explores how family cohesion is related to adolescent self-esteem, including the mediating role of interpersonal self-support and the moderating role of school attachment.

### 1.1 Family cohesion and self-esteem

Conceptualizations of family functioning have been significantly advanced by foundational theorists, including Beavers (13), Olson (6), and Shek (14), who operationalize this construct through measurable systemic characteristics. Olson’s framework posits family cohesion and family adaptability as dual structural dimensions of family functioning. Within this paradigm, family cohesion denotes the strength and quality of emotional bonds, mutual support, and interdependence among members, reflecting both the affective unity and functional solidarity within familial relational systems. Reviews of the family-functioning literature further distinguish structural and process-oriented approaches to this construct (15).

The second category is represented by Epstein and Skinner (16, 17), who suggest that family members should rely on each other to complete shared tasks and solve problems, thereby promoting the physical and mental health of family members. The present study adopts Olson’s definition of family cohesion to explore its relationship with self-esteem. Self-esteem refers to people’s attitudes and evaluations of their abilities and self-worth. According to Erikson’s theory of personality development (18), adolescent students are in the critical period of self-identity formation, where their developmental task is to establish identity and prevent identity confusion. During this period, individuals may experience negative psychological states, interpersonal indifference, and even antisocial tendencies (19).

Studies have shown that when parents adopt a positive parenting style, family relationships are more intimate and the family atmosphere more harmonious, children’s successful experiences in learning and life are reinforced, which is conducive to the formation of positive self-awareness and high self-esteem (20). Based on the theory and empirical studies above, it is reasonable to propose that good family cohesion is associated with higher levels of adolescent self-esteem. Thus, hypothesis H1 is proposed: family cohesion is positively associated with self-esteem.

### 1.2 The mediating effect of interpersonal self-support

Self-support has been regarded as an important personality trait or moral character since ancient times. In Huang Xiting’s theory, the self-supporting personality includes two dimensions: interpersonal self-support and personal self-support. Interpersonal self-support refers to personality characteristics formed by individuals in solving basic interpersonal problems, including five traits: interpersonal independence, interpersonal initiative, interpersonal responsibility, interpersonal flexibility, and interpersonal openness (21). Interpersonal self-support can integrate individuals’ psychological factors and coordinate the balance between self-support and dependence on the environment and others (22). Familial influences constitute a critical substratum of subjective psychological well-being, with family cohesion functioning as a core mechanism through which collectivist-oriented support systems facilitate the development of interpersonal self-support. Specifically, empirical research indicates that parent-child interaction quality and parental dyadic cohesion show significant associations with children’s self-support behaviors, partially mediated by school attachment (23). It has been shown that family cohesion is positively related to self-support (24). Self-support is a comprehensive personality factor; although it does not belong to the basic personality dimensions, it is an important part of a sound personality. Previous studies showed that self-esteem and self-support have a significant positive correlation (12, 25). In summary, empirical evidence underscores significant interconnections among family cohesion, interpersonal self-support, and self-esteem. However, scholarly investigations have yet to systematically interrogate the triadic relationship among these constructs. Grounded in extant theoretical frameworks and empirical research, the present study advances H2: interpersonal self-support mediates the association between family cohesion and self-esteem.

### 1.3 The moderating effect of school attachment

For teenagers, school is the most important setting besides family for living and study. Following Mouton et al. (26), school attachment refers to students’ affinity with the school environment, developed through participation in school activities and through support from peers and school staff. Beyond a sense of warmth toward the school, it also includes perceiving respect and support as an independent individual (27). School attachment is an important aspect of the sense of school belonging (28) and is positively associated with adolescents’ mental health (29). Adolescents raised in cohesive familial environments benefit from parents who engage with their offspring’s educational trajectories and psychosocial skill acquisition. Such caregivers prioritize cultivating capacities for autonomous reasoning, peer relationship management, and navigating complex social challenges, thereby fostering robust affective bonds between adolescents and their educational institutions. This relational scaffolding establishes foundational conditions for positive school attachment to emerge as both an academic resource and a psychological safeguard (30). Empirical research has consistently demonstrated that family cohesion is positively associated with school belonging (31, 32). According to developmental systems theory, individuals and their environment form an integrated system in which individual-level subsystems (e.g., biology, cognition, emotion) and environmental-level subsystems (e.g., interpersonal relationships, physical environment, social systems) mutually influence each other (33). Some studies indicate that cohesion and conflict in the teacher-child relationship directly affect children’s socialization and school adjustment, suggesting that relationship quality in the school environment can modulate the influence of the family environment on individual development (34).

Integrating family system theory and ecological-developmental perspectives, the present study proposes that school attachment may function as a moderator, specifically of the first stage of the mediation pathway (family cohesion → interpersonal self-support). This specification is grounded in the rationale that school attachment, as an external relational resource, primarily enhances the individual’s capacity to internalize familial support into autonomous interpersonal competencies (i.e., interpersonal self-support), rather than directly altering the subsequent link from interpersonal self-support to self-esteem, which is more internally driven by self-evaluation processes. It should be noted that this first-stage specification is a theoretical assumption: because only first-stage moderation was tested in this study, the exclusivity of this location relative to alternative moderation points (e.g., the second stage) was not empirically established. Consequently, the study posits H3: school attachment moderates the first stage of the mediation pathway linking family cohesion to self-esteem through interpersonal self-support, such that the positive association between family cohesion and interpersonal self-support is stronger for adolescents with higher school attachment.

In summary, this investigation examines the association between family cohesion and adolescent self-esteem, with explicit focus on its dual mechanisms of mediation and moderation. Building on established theoretical foundations, the study advances three hypotheses: (H1) family cohesion is positively associated with self-esteem; (H2) interpersonal self-support mediates this association; and (H3) the indirect association is moderated by school attachment. Synthesizing H1, H2, and H3 constitutes a moderated mediation framework in which school attachment moderates the first stage of the indirect pathway linking family cohesion to self-esteem via interpersonal self-support. The proposed conceptual model is presented in **Figure 1**.

**Figure 1:**
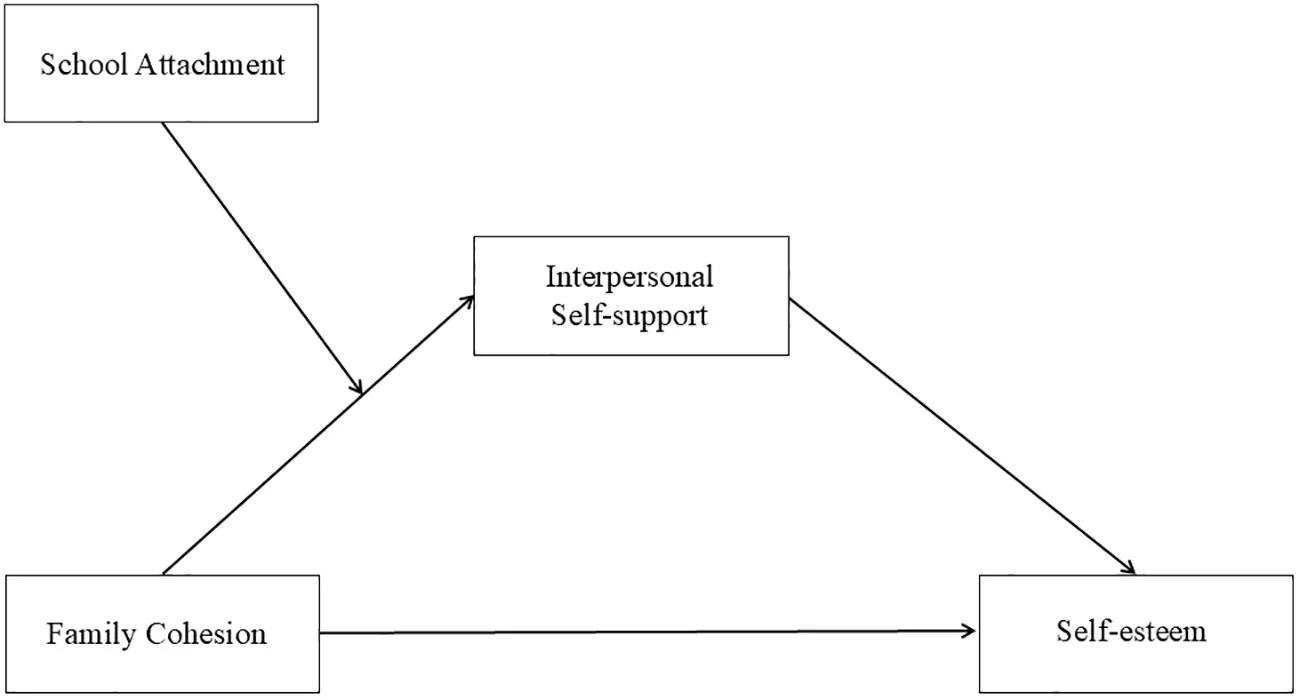
Hypothetical model graph.

## 2 Methods

### 2.1 Participants and procedure

A cross-sectional survey design was employed in this study. A sample of *n* = 728 adolescents was recruited from five public junior high schools located in Hunan Province, China, using a cluster sampling strategy (classes sampled within schools). In terms of grade level, 33.38% of participants were in the first grade of junior high school, 42.03% in the second grade, and 24.59% in the third grade. The age of the participants ranged from 12 to 16 years, with a mean age of 13.81 years (*SD* = 0.91). The sample consisted of 320 male students (44.0%) and 408 female students (56.0%).

Data collection was conducted on a class-by-class basis during regular school hours by trained research assistants. A total of 889 questionnaires were distributed; 161 invalid questionnaires (e.g., blank or incomplete responses) were removed, leaving 728 valid questionnaires for analysis (valid response rate = 81.9%). Prior to administering the questionnaires, written informed consent was obtained from the school administrators, class teachers, participants, and the participants’ parents or legal guardians, and the adolescents provided their own assent. Participation was entirely voluntary, and adolescents were assured of the strict anonymity and confidentiality of their responses. The survey packets were completed independently within approximately 20 minutes. There were no missing data in the final analyzed dataset. The study was approved by the Academic Ethics Committee of Hunan University of Arts and Sciences.

### 2.2 Measures

#### 2.2.1 Family cohesion

The family cohesion scale comprised the 16-item family cohesion subscale of the Chinese revised version of the Family Adaptability and Cohesion Evaluation Scales (FACES II-CV; 35), based on the original FACES II (36). For example, “in times of difficulty, family members will do their best to support each other” (37). The 5-point Likert response format ranged from 1 (“no”) to 5 (“always”), and items 2, 5, 10, and 15 were reverse-scored, following the original FACES II item numbering. Higher scores indicate better cohesion between family members. The scale showed good internal consistency in this study (Cronbach’s α = 0.914). A confirmatory factor analysis (CFA) was conducted with Mplus 8.3: χ²/*df* = 4.06, CFI = .923, TLI = .911, RMSEA = .065, indicating acceptable structural validity.

#### 2.2.2 Self-esteem

The Self-Esteem Scale (SES; 2) comprises ten items rated on a four-point Likert-type response format (1 = very consistent to 4 = very inconsistent), such as “I feel that I am a valuable person, at least on the same level as others.” Following common practice for Chinese adolescent samples, item 8 was excluded, leaving nine items. Given the response anchors, the five positively worded items were reverse-scored, whereas the four negatively worded items were scored in their original direction, so that higher scores indicate higher self-esteem. Item scores were averaged to form a composite index (range 1–4), and the item-level means reported in **Table 1** are the scores used in the analyses. The scale showed good internal consistency (Cronbach’s α = 0.90). A CFA conducted with Mplus 8.3 yielded χ²/*df* = 4.04, CFI = .938, TLI = .918, RMSEA = .075, indicating acceptable structural validity.

**Table 1:** Descriptive statistics and intercorrelations among study variables (n = 728).

Variables	*Mean*	*SD*	1	2	3	4
1. Family cohesion	3.62	0.749	1			
2. Self-esteem	3.002	0.582	0.580***	1		
3. self-support	3.569	0.509	0.501***	0.521***	1	
4. School attachment	4.022	0.842	0.534***	0.490***	0.383***	1

*p <0.05, **p <0.01, ***p <0.001, the same below.

#### 2.2.3 Interpersonal self-support

The self-supporting personality scale for adolescent students was developed by Xia and Huang (38); the interpersonal self-support subscale was used in this study. This subscale has 20 items, such as “Talking to strangers alone is easy to panic.” It operationalizes interpersonal self-support through five interrelated dimensions: interpersonal independence, interpersonal initiative, interpersonal responsibility, interpersonal flexibility, and interpersonal openness, scored on a 5-point Likert scale, with higher composite scores reflecting greater relational competence. Cronbach’s α was 0.801. A CFA conducted with Mplus 8.3 yielded χ²/*df* = 2.90, CFI = .893, TLI = .867, RMSEA = .051. Because the CFI and TLI values fall below the conventional.90 threshold, the structural validity of this scale, which measures the mediator, is not fully supported. We therefore interpret the mediating-effect estimates with caution and examine the implications of this measurement limitation in Section 5.

#### 2.2.4 School attachment

The school attachment dimension of the Psychological Sense of School Membership Scale (PSSM; 27), Chinese version (39), was used. The subscale comprised items 13, 16, and 17 of the full 18-item PSSM, such as “I am proud to belong to this school,” scored on a 5-point Likert scale (1 = never, 5 = always). Item 16 was reverse-scored, consistent with the original scale’s scoring instructions. Higher scores indicate stronger school attachment. In the current sample, Cronbach’s α was 0.72 and McDonald’s ω was 0.74, indicating acceptable but modest internal consistency for a three-item scale. A confirmatory factor analysis of the three items showed that all standardized factor loadings were significant (*p* <.001) and exceeded.50 (40), and the subscale correlated strongly with the total PSSM score (*r* = .824, *p* <.001; note that this correlation is partially circular because the three items are part of the total scale). Given the modest internal consistency, the school attachment results should be interpreted with some caution.

### 2.3 Statistical analysis

Confirmatory factor analyses were conducted with Mplus 8.3 to evaluate the structural validity of each scale and the overall four-factor measurement model. Because the indicators were ordinal (4- or 5-point Likert scales), the overall four-factor measurement model was additionally estimated with the WLSMV estimator, which accounts for the categorical nature of the indicators. The moderated mediation model was tested with Hayes’ PROCESS macro (Model 7; 41) in SPSS 27 using observed variables, with 5,000 bootstrap samples; all predictor variables were standardized prior to analysis. The first-stage model regressed interpersonal self-support on family cohesion, school attachment, and their interaction, and the second-stage model regressed self-esteem on family cohesion and interpersonal self-support.

Because participants were clustered within classes and schools, the independence assumption of the observed-variable path analysis may be violated. Age, gender, and grade were not included as covariates in the primary analyses, although re-estimating the model with these covariates yielded virtually identical results (see Section 3.3); family socioeconomic status was not assessed, and the reported estimates should therefore be read as unadjusted associations. Finally, a sensitivity power analysis (42) indicated that, with *n* = 728, α = .05, and three predictors in the first-stage model, the design could detect a moderation effect of *f*² = .011 (equivalent to Δ*R*² = .008) with 80% power. The observed interaction therefore falls at the boundary of what this design could detect with conventional power, which we take into account when interpreting its practical significance.

## 3 Results

### 3.1 Common method bias

To address potential common method bias, procedural safeguards, including anonymized response protocols, were implemented (43, 44). Harman’s single-factor test extracted multiple factors with eigenvalues exceeding unity, with the first factor accounting for 23.24% of total variance, below the conventional 40% threshold. When the ordinal nature of the indicators was accounted for with the WLSMV estimator, the model fit well (CFI = .916, TLI = .913, RMSEA = .049, SRMR = .057).

### 3.2 Descriptive statistics and correlations

Means, standard deviations, and intercorrelations among the study variables are shown in **Table 1**. Family cohesion was positively associated with self-esteem, interpersonal self-support, and school attachment.

### 3.3 Moderated mediation analysis

To test the moderated mediation model, we used Model 7 of Hayes’ PROCESS macro (Version 4.1; 41) with 5,000 bootstrap samples. This approach evaluates the direct and indirect pathways while testing whether school attachment moderates the first stage of the mediation pathway (family cohesion → interpersonal self-support). All predictor variables were standardized prior to analysis. The results of the regression analyses are presented in **Table 2**.

**Table 2:** Results of the moderated mediation analysis.

Item	*b*	*SE*	*t*	*p*	95% CI
Model 1 (outcome: interpersonal self-support)					
Constant	−0.041	0.035	−1.176	.240	[−0.108, 0.027]
Family cohesion (X)	0.424	0.037	11.311	<.001	[0.350, 0.497]
School attachment (W)	0.189	0.039	4.909	<.001	[0.114, 0.265]
Family cohesion × school attachment	0.076	0.026	2.895	.004	[0.024, 0.128]
Model fit	*R*² = .278; *F* = 93.149; *p* <.001				
Interaction effect change	Δ*R*² = .008; *F* = 8.379; *p* = .004				
Model 2 (outcome: self-esteem)					
Constant	0.000	0.029	0.000	1.000	[−0.056, 0.056]
Family cohesion (X)	0.426	0.033	12.901	<.001	[0.362, 0.491]
Interpersonal self-support (M)	0.307	0.033	9.282	<.001	[0.242, 0.372]
Model fit	*R*² = .407; *F* = 248.961; *p* <.001				

Conditional indirect effects and index of moderated mediation					
Conditional indirect effect (family cohesion → interpersonal self-support → self-esteem)	Effect	Boot *SE*	Boot 95% CI		
Low school attachment (−1 *SD*)	0.107	0.021	[0.068, 0.150]		
Mean school attachment (0 *SD*)	0.130	0.020	[0.094, 0.170]		
High school attachment (+1 *SD*)	0.153	0.024	[0.109, 0.201]		
Index of moderated mediation: school attachment (W)	0.023	0.011	[0.004, 0.045]		

B, standardized regression coefficient; SE, standard error; CI, confidence interval; 5,000 bootstrap samples were used for the conditional indirect effects and the index of moderated mediation.

First, we examined the model predicting interpersonal self-support. The overall model was statistically significant, *R*² = .278, *F* = 93.15, *p* <.001. Standardized family cohesion was positively associated with interpersonal self-support (*b* = 0.424, *SE* = 0.037, *t* = 11.31, *p* <.001, 95% CI [0.350, 0.497]). Standardized school attachment was also positively associated with interpersonal self-support (*b* = 0.189, *SE* = 0.039, *t* = 4.91, *p* <.001, 95% CI [0.114, 0.265]). The interaction between family cohesion and school attachment significantly predicted interpersonal self-support (*b* = 0.076, *SE* = 0.026, *t* = 2.89, *p* = .004, 95% CI [0.024, 0.128]). The interaction term explained an additional 0.8% of the variance in interpersonal self-support, Δ*R*² = .008, *F* = 8.38, *p* = .004.

Simple slope analysis (**Figure 2**) showed that the positive association between family cohesion and interpersonal self-support was significant at all levels of school attachment but grew stronger as school attachment increased. Specifically, the association was positive and significant at low (−1 *SD*: *b* = 0.348, *SE* = 0.044, *t* = 7.89, *p* <.001, 95% CI [0.261, 0.434]), moderate (0 *SD*: *b* = 0.424, *SE* = 0.037, *t* = 11.31, *p* <.001, 95% CI [0.350, 0.497]), and high (+1 *SD*: *b* = 0.500, *SE* = 0.047, *t* = 10.54, *p* <.001, 95% CI [0.407, 0.593]) levels of school attachment. The moderation therefore reflects a difference in degree rather than the presence versus absence of the association.

**Figure 2:**
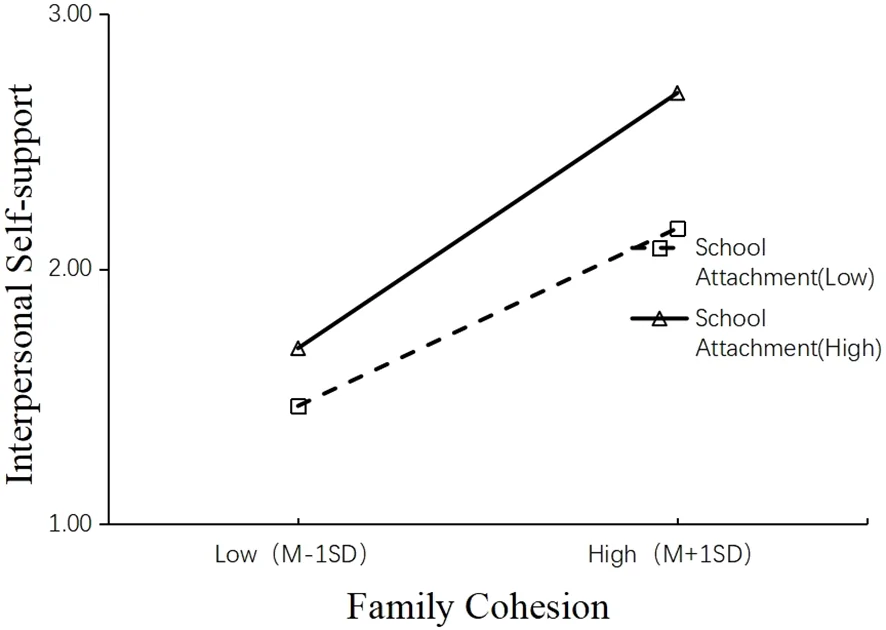
School attachment moderating the association between family cohesion and interpersonal self-support.

Second, we examined the model predicting self-esteem. The model was significant, *R*² = .407, *F* = 248.96, *p* <.001. Both family cohesion (*b* = 0.426, *SE* = 0.033, *t* = 12.90, *p* <.001, 95% CI [0.362, 0.491]) and interpersonal self-support (*b* = 0.307, *SE* = 0.033, *t* = 9.28, *p* <.001, 95% CI [0.242, 0.372]) were positive and significant predictors of self-esteem. The direct effect of family cohesion on self-esteem remained significant (*b* = 0.426, *SE* = 0.033, *t* = 12.90, *p* <.001), supporting partial mediation.

To evaluate the moderated mediation hypothesis, we examined the conditional indirect effects of family cohesion on self-esteem via interpersonal self-support across levels of school attachment. The indirect effect was positive and statistically significant at low (−1 *SD*: Effect = 0.107, Boot *SE* = 0.021, 95% Boot CI [0.068, 0.150]), mean (0 *SD*: Effect = 0.130, Boot *SE* = 0.020, 95% Boot CI [0.094, 0.170]), and high (+1 *SD*: Effect = 0.153, Boot *SE* = 0.024, 95% Boot CI [0.109, 0.201]) levels of school attachment.

Finally, the Index of Moderated Mediation was positive and statistically significant (Index = 0.023, Boot *SE* = 0.011, 95% Boot CI [0.004, 0.045]). Because the 95% bootstrap confidence interval did not include zero, H3 was supported: the indirect association between family cohesion and adolescent self-esteem through interpersonal self-support was stronger at higher levels of school attachment. Given the small magnitude of the index and of the incremental variance explained by the interaction (Δ*R*² = .008), this moderation should be interpreted as a modest, degree-based effect. As a robustness check, the model was re-estimated with age, gender, and grade included as covariates; the first-stage interaction (*b* = 0.082, *SE* = 0.026, *p* = .004; Δ*R*² = .010) and the index of moderated mediation (Index = 0.025, 95% bootstrap CI [0.005, 0.047]) remained statistically significant, and the conditional indirect effects were 0.107 (low), 0.132 (mean), and 0.157 (high school attachment).

## 4 Discussion

### 4.1 Family cohesion and self-esteem

This study found that family cohesion was positively associated with adolescent self-esteem. This is consistent with the Circumplex Model of Family Functioning and with prior studies (5, 6, 45). According to this model, families with high cohesion can enhance teenagers’ sense of self-worth by providing emotional support and a sense of belonging. Specifically, the close emotional connection between family members makes teenagers feel accepted and valued, and this positive feedback may be transformed into a stable self-esteem experience through the formation of “self-identity” proposed by Erikson (18). In addition, in environments with high family cohesion, parents tend to adopt warm, encouraging parenting styles that help adolescents accumulate successful experiences and strengthen their self-efficacy. This result suggests that family interventions should focus on improving the quality of parent-child communication, for example by enhancing emotional connection among members through family group activities or psychological education courses, to provide basic support for the development of adolescent self-esteem.

### 4.2 The mediating effect of interpersonal self-support

This investigation provides evidence that interpersonal self-support partially mediates the family cohesion–self-esteem association among adolescents. The observed effects indicate that familial relational harmony is not only directly associated with self-esteem but is also related to self-esteem through the cultivation of interpersonal self-support competencies in nurturing family environments. These competencies are in turn associated with more positive self-appraisals, establishing a dual pathway through which family systems may relate to adolescent self-esteem. First, a close family usually provides emotional support, understanding, and encouragement, and individuals can learn to think independently, solve problems, and take responsibility in this environment. Therefore, good family cohesion may help cultivate an interpersonal self-supporting personality, consistent with previous studies (46). According to the self-supporting personality theory (21, 22), interpersonal self-support develops as individuals repeatedly exercise interpersonal initiative, responsibility, flexibility, and openness within supportive relationships; family cohesion provides the secure relational context in which such practice is possible. This account is also consistent with attachment and social-learning perspectives, which hold that warm, secure family bonds provide models and opportunities for adolescents to acquire and rehearse interpersonal competencies that generalize to broader social contexts. The principal contribution of this investigation is the integration of interpersonal self-support into the mediation pathway linking family functioning to self-esteem, offering a competence-mediated account of the family–individual linkage.

It is important to place the magnitude of these effects in context. The indirect effect of family cohesion on self-esteem via interpersonal self-support ranged from 0.107 to 0.153 across levels of school attachment, whereas the direct effect of family cohesion on self-esteem (*b* = 0.426) was substantially larger. The indirect pathway therefore represents a secondary, complementary route rather than the dominant mechanism, and its practical significance should be interpreted accordingly. Practically, these findings suggest that programs aiming to enhance adolescent self-esteem should primarily strengthen family cohesion while also providing opportunities (e.g., scenario-based role-playing, social-navigation workshops) for adolescents to develop interpersonal self-support.

### 4.3 The moderating effect of school attachment

The present study indicates that school attachment moderates the first stage of the association between family cohesion and self-esteem via interpersonal self-support. The positive association between family cohesion and interpersonal self-support was significant at all levels of school attachment but was stronger among adolescents with higher school attachment. This result is consistent with an interactive view of ecological systems theory: family and school, as core elements of the microsystem, jointly shape adolescent psychological development (47).

Theoretically, school attachment was positioned as a first-stage moderator because it provides external relational resources (belonging, validation, role models) that may enhance the internalization of family-based emotional support into autonomous interpersonal competencies, whereas the direct path from family cohesion to self-esteem and the second-stage path from interpersonal self-support to self-esteem are more rooted in intra-familial dynamics and intra-individual self-evaluation. This specification is consistent with prior research indicating that external environmental factors such as school belonging primarily interact with family processes at the level of skill acquisition and behavioral modeling (31, 32). However, because only first-stage moderation (PROCESS Model 7) was tested, alternative moderation locations cannot be ruled out (e.g., a second-stage model in which school attachment moderates the path from interpersonal self-support to self-esteem). The first-stage specification should therefore be regarded as a theoretically derived assumption rather than an empirically established exclusivity.

In terms of magnitude, the interaction accounted for only 0.8% of additional variance in interpersonal self-support (Δ*R*² = .008), and the Index of Moderated Mediation was small (0.023). The sensitivity power analysis further indicated that effects of this size fall at the boundary of what the present design could detect with 80% power. The moderation should therefore be interpreted as a modest difference in the strength of the first-stage association, and replications in larger samples are warranted before drawing strong practical conclusions. For example, students with a good teacher-student relationship may transfer social strategies learned in the family to the school situation, forming a “family-school” synergistic effect. Schools may nonetheless reasonably seek to strengthen teacher-student interaction and peer relationships, for example through cooperative learning projects or mental-health-themed activities, to enhance students’ sense of belonging and optimize the psychological benefits of family cohesion.

## 5 Limitations and future directions

Empirical findings indicate that family cohesion was positively associated with adolescent self-esteem. Families with good cohesion may enhance teenagers’ sense of self-worth and security by providing emotional support and a sense of belonging. This finding highlights the central role of the family in adolescent mental health and suggests the importance of family education and family relationships for adolescent self-esteem. Furthermore, the analysis identified interpersonal self-support as a significant mediator of the association between family cohesion and self-esteem. Good family cohesion may promote adolescent interpersonal self-support, and higher interpersonal self-support is in turn associated with higher self-esteem. This result suggests that family education should also focus on cultivating adolescents’ autonomy and confidence in interpersonal communication. Adolescents with high school attachment showed a stronger association between family cohesion and self-esteem through interpersonal self-support, underscoring the importance of schools in adolescent psychological development. Schools should create inclusive and supportive environments to enhance students’ sense of belonging and thereby support their mental health and self-esteem.

Several limitations should be acknowledged. First, the cross-sectional, single-time-point design precludes conclusions about temporal precedence or causal direction. Alternative directional models remain plausible; for example, higher self-esteem may lead to more positive perceptions of family cohesion, or self-supporting individuals may actively cultivate closer family interactions, and reciprocal or cyclical effects cannot be ruled out. Longitudinal or experimental designs are needed to discriminate among these alternatives. Second, although procedural safeguards and the confirmatory factor analytic comparisons provide some evidence against common method bias, the data are self-reported, and an unmeasured latent method factor model was not estimated. Third, participants were sampled in clusters, and the primary analyses did not adjust for this clustering or for covariates such as age, gender, and grade; re-estimating the model with these covariates yielded virtually identical results, but future analyses should nonetheless use multilevel models or cluster-robust standard errors. Finally, the sample was drawn from five junior high schools in Hunan Province, limiting generalizability, and the measures relied on self-report, which may be subject to social-desirability and response biases. Future studies should expand the sample to cover different regions and cultural backgrounds and combine multiple measurement methods, such as tracking surveys and interviews, to improve the reliability and validity of the data.

## 6 Conclusions

This research examined the association between family cohesion and adolescent self-esteem, with interpersonal self-support as a mediator and school attachment as a moderator. The findings indicate that family cohesion is positively associated with self-esteem, consistent with theoretical frameworks such as Erikson’s identity formation theory and the Circumplex Model of Family Functioning. Interpersonal self-support partially mediated this association, and school attachment moderated the first stage of the mediation pathway, with a stronger first-stage association among adolescents reporting higher school attachment. Because the design was cross-sectional and self-reported, these associations should not be interpreted causally.

Practically, these findings call for collaborative efforts between families and schools to support adolescents’ self-esteem. Families should prioritize emotional support and autonomy encouragement, while schools should strengthen teacher-student relationships and peer engagement to create supportive learning environments. Such joint initiatives may optimize the benefits of family and school influences, ultimately promoting mental health and adaptive development among adolescents.

## Data Availability

The raw data supporting the conclusions of this article will be made available by the authors, without undue reservation.
